# Potential Assessment of UGT2B17 Inhibition by Salicylic Acid in Human Supersomes In Vitro

**DOI:** 10.3390/molecules26154410

**Published:** 2021-07-21

**Authors:** Hassan Salhab, Declan P. Naughton, James Barker

**Affiliations:** School of Life Sciences, Pharmacy and Chemistry, Kingston University, Kingston-Upon-Thames, London KT1 2EE, UK; d.naughton@kingston.ac.uk (D.P.N.); j.barker@kingston.ac.uk (J.B.)

**Keywords:** glucuronidation, human supersomes, salicylic acid, UGT2B17, testosterone

## Abstract

Glucuronidation is a Phase 2 metabolic pathway responsible for the metabolism and excretion of testosterone to a conjugate testosterone glucuronide. Bioavailability and the rate of anabolic steroid testosterone metabolism can be affected upon UGT glucuronidation enzyme alteration. However, there is a lack of information about the in vitro potential assessment of UGT2B17 inhibition by salicylic acid. The purpose of this study is to investigate if UGT2B17 enzyme activity is inhibited by salicylic acid. A UGT2B17 assay was developed and validated by HPLC using a C18 reversed phase column (SUPELCO 25 cm × 4.6 mm, 5 μm) at 246 nm using a gradient elution mobile phase system: (A) phosphate buffer (0.01 M) at pH = 3.8, (B) HPLC grade acetonitrile and (C) HPLC grade methanol. The UGT2B17 metabolite (testosterone glucuronide) was quantified using human UGT2B17 supersomes by a validated HPLC method. The type of inhibition was determined by Lineweaver–Burk plots. These were constructed from the in vitro inhibition of salicylic acid at different concentration levels. The UGT2B17 assay showed good linearity (R2 > 0.99), acceptable recovery and accuracy (80–120%), good reproducibility and acceptable inter and intra-assay precision (<15%), low detection (6.42 and 2.76 μM) and quantitation limit values (19.46 and 8.38 μM) for testosterone and testosterone glucuronide respectively, according to ICH guidelines. Testosterone and testosterone glucuronide were found to be stable up to 72 h in normal laboratory conditions. Our investigational study showed that salicylic acid uncompetitively inhibited UGT2B17 enzyme activity. Thus, drugs that are substrates for the UGT2B17 enzyme have negligible potential effect of causing interaction with salicylic acid in humans.

## 1. Introduction

By definition, UGT enzymes are glycoproteins, i.e., Phase 2 drug-mediated enzymes that can glucuronidate biologically anabolic steroids, such as testosterone, xenobiotic and phenolic compounds, into more lipophobic molecules [1,2]. UGT enzymes are found in the endoplasmic reticulum [3]. After transforming xenobiotic substances, fatty acid derivatives or steroids into hydrophilic group moieties, they are easily excreted by the liver or bile [4]. These biological transformations are known as conjugation reactions [5] and are mediated by various processes, such as amino acid, glutathione conjugation, sulfation, acetylation, glucuronidation and methylation [6].

The glucuronidation reaction accounts for a major pathway in the Phase 2 drug-mediated process [4]. The reason for its importance is that it is involved in the metabolism of about 40–70% of clinical drugs in humans. UDP-glucuronosyltransferases are Phase 2 mediated enzymes responsible for the breaking-down of glucuronic acid from UDP-glucuronic acid, which occurs mainly in the liver and small intestine [7]. The obtained metabolites exhibit increased water solubility due to their polarity [7]. Up till the present, only four UGT families have been discovered in humans. These include UGT1, UGT2, UGT3 and UGT8 [7].

**Figure 1:**
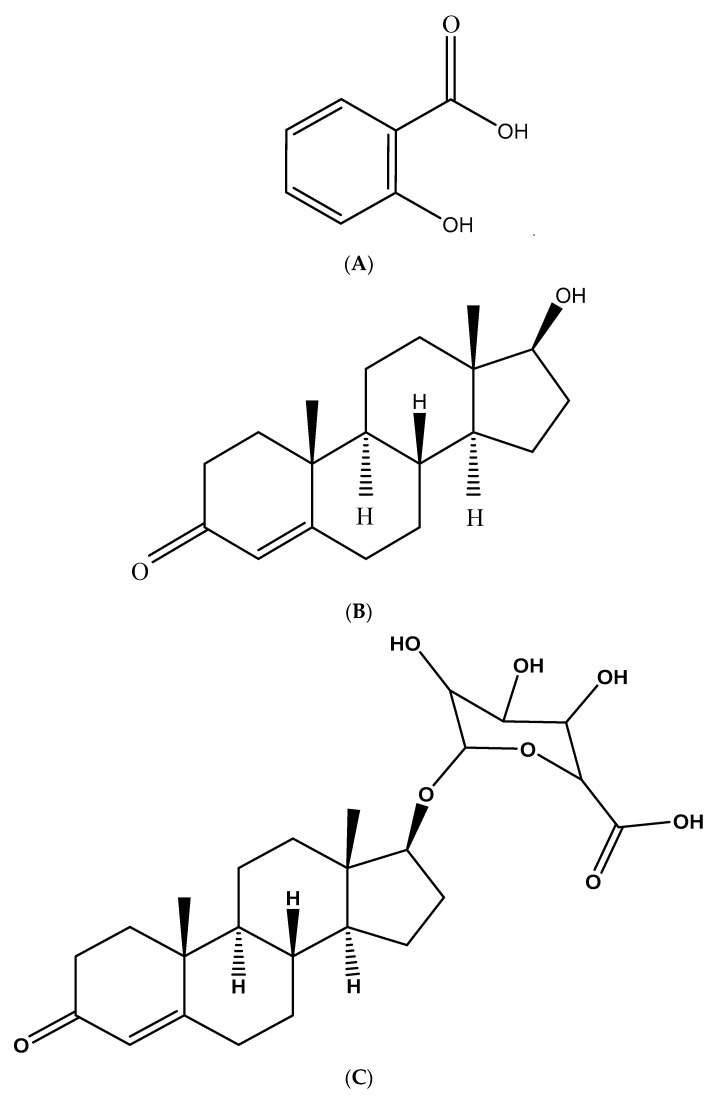
Chemical structures of: salicylic acid (**A**), testosterone (**B**), and testosterone glucuronide (**C**).

The UGT2B17 enzyme, along with the UGT2B15 enzyme, is responsible in glucuronidating testosterone (Figure 1B) to a conjugate testosterone glucuronide (testosterone b-D-glucuronide) (Figure 1C) [3]. The UDP-glucuronosyltransferase UGT2B17 enzyme possesses more than double the glucuronidation activity compared to the UGT2A1 enzyme that can glucuronidate the anabolic hormone testosterone [2]. Previous studies have demonstrated that testosterone metabolism differs between individuals and alters with ethnicity due to a variety in UGT2B17 expression [1]. Investigational in vitro studies have shown that the rate of testosterone glucuronidation can be decreased by UGT2B17 inhibitors such as anti-inflammatory drugs [1]. Moreover, various drugs inhibit UGT2B17 enzyme activity, which is glucuronidated as a substrate [1]. Until now, little is known about the in vitro potential assessment of UGT2B17 enzyme activity inhibition by salicylic acid in human supersomes. Human supersome is a recombinant enzyme containing human UGT2B17 cDNAs and is prepared from insect cells infected with baculovirus [1].

Interestingly, UGT1A1 exhibits two activities; a low activity in coumarin conjugation and a moderate activity in flavanone and steroid conjugation [4]. Opioids conjugate primarily by the UGT2B7 enzyme only; however, carboxylic acids conjugate by UGT1A3, UGT1A9 and UGT2A1.

Previous studies have shown that UGT1A10 was the only key isozyme responsible for the glucuronidation of bioflavonoids [4]. Other co-workers have found that UGT1A9 and UGT1A3 are the two UGT isoforms involved in the glucuronidation of flavonoids. UGT inhibition alters the hormone levels that can cause cancer [8]. For example, various bioactive components (green and black tea powder, cacao, quercetin, allspice and silymarin) have the potency to inhibit UGT enzyme activity, but further investigations need to be carried out in vivo to evaluate their effect clinically.

Alteration in the phytochemicals’ effect with a concurrent decrease in sensitivity of enzyme activity occurs due to the metabolism of phytochemicals by UDP-glucuronosyltransferases [8]. For example, flavonoid compounds are bound with sulfate and glucuronide; thus, alteration in the efficacy of these can be produced by either UGT or sulfotransferase polymorphisms [5]. Foodstuffs such as cruciferous vegetables, resveratrol and citrus have the potency to induce UGT enzymes. In contrast, other bioactive compounds, including honeybush tea, curcumin, rooibos, astaxanthin, soy and ferulic acid, can enhance UGT enzyme activity in animal studies [5].

Recently, in vitro studies have showed that white and green teas, catechin constituents and red wine inhibit the UGT2B17 enzyme activity in human supersomes [3]. Salicylic acid (Figure 1A) (carboxylic acid functionality), usually formed as a metabolite of acetylsalicylic acid (aspirin), possesses many beneficial pharmacological activities, such as anti-inflammatory, antioxidant, and vasodilator effects [9]. Given the in vitro potential role of red wine and white and green teas in the suppression of UDP-glucuronosyltransferase UGT2B17 mediated testosterone glucuronidation, it is timely to investigate the potential inhibition effect of salicylic acid in human supersomes on UGT2B17 enzyme activity.

Our previous study shown that CYP2C11 enzyme activity can be inhibited non-competitively by salicylic acid [10]. In addition, our previous work proved that CYP2E1 enzyme activity could be inhibited competitively and non-competitively by salicylic acid [11]. Thus, the purpose of this investigational study was to evaluate the potential UGT2B17 enzyme activity inhibition by salicylic acid (Figure 1A) in vitro.

## 2. Results and Discussion

### 2.1. UGT2B17 Assay Analytical Wavelength Selection

According to our previous investigational experiment, salicylic acid has a maximum absorption band at 195 nm [11]. Based on the Jenkinson et al. (2012) study, a modified wavelength of 243 nm was chosen for the UGT2B17 assay.

### 2.2. UGT2B17 Assay Method Development

The UGT2B17 assay was developed at a flow rate of 1 mL/min and a temperature of 25 °C using a C18 (SUPELCO 25 cm × 4.6 mm, 5 µm) and HPLC gradient mode elution programming as stated in Table 1.

### 2.3. UGT2B17 Assay Method Validation

#### 2.3.1. Selectivity and Specificity

**Figure 2:**
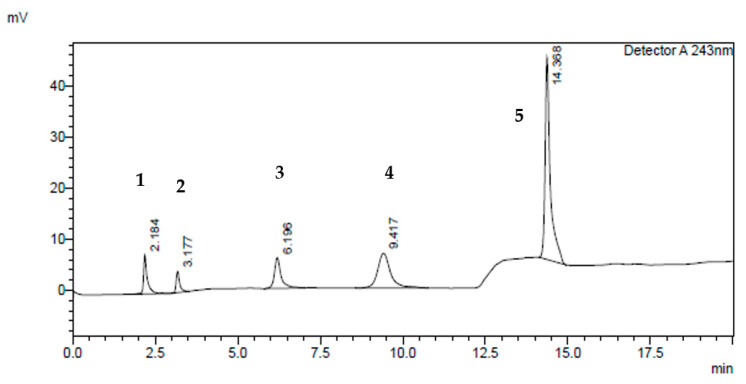
Typical HPLC chromatogram of UGT2B17 components at 243 nm using gradient elution programming. The peaks marked are: (**1**) UGT2B17 enzyme (Rt = 2.184 min); (**2**) salicylic acid (100 µM) (Rt = 3.177 min); (**3**) phenacetin (50 µM) (Rt = 6.196 min); (**4**) testosterone glucuronide (100 µM) (Rt = 9.417 min); and (**5**) testosterone (200 µM) (Rt = 14.388 min), respectively.

The optimum mobile phase composition was chosen using a gradient elution programming system (Figure 2) that resulted in a nicely separated salicylic acid peak from the UGT2B17 metabolite (testosterone glucuronide), phenacetin, testosterone and UGT2B17 enzyme peaks at T = 25 °C using C18 (SUPELCO 25 cm × 4.6 mm, 5 µm), at 1 mL/min flow rate, and wavelength of λ = 243 nm.

#### 2.3.2. Robustness Tests

##### Flow Rate Alteration

(**A**) A robustness test was carried out by decreasing the flow rate by 0.2 mL/min. The effect on both retention time and peak area was evaluated for each component in the UGT2B17 assay. As 1 mL/min was the optimum flow rate for HPLC, 0.8 mL/min was chosen, as it is ±0.2 mL/min. Table 2 demonstrates the retention time and peak area of UGT2B17 components by the effect of flow rate variation.

An ANOVA test with a single factor analysis was carried out to investigate the effect of flow rate on the peak area of each component. Statistically, no significant difference was evidenced between the peak area of each component at two different flow rates (*p*-value = 0.095 > 0.01) and a variance of 0.011 with the significance level set at 0.05. However, phenacetin, testosterone glucuronide and testosterone elute later than when the flow rate was 1 mL/min.

(**B**) A further robustness test was carried out by increasing the flow rate by 0.2 mL/min. The effect on both retention time and peak area was again evaluated for each component in the UGT2B17 assay. As 1 mL/min was the optimum flow rate for HPLC, thus, 1.2 mL/min was selected, as it is ±0.2 mL/min. Table 3 demonstrates the retention time and peak area of UGT2B17 components and the effect of flow rate variation.

An ANOVA test with a single factor analysis was carried out for the effect of flow rate on the peak area of each component. Statistically, a slightly significant difference was evidenced between the peak area of each component at two different flow rates (*p*-value = 0.006) and a variance of 0.011 with the significance level set at 0.05. Thus, a 1 mL/min flow rate was chosen as the optimized parameter on HPLC, noting that the resolution between UGT2B17 enzyme and salicylic acid peaks (1 min) was greater than the difference in retention time between salicylic acid and UGT2B17 enzyme peaks (0.81 min) with a flow rate of 1.2 mL/min. Therefore, the UGT2B17 assay was considered to be a robust method based upon flow rate variation.

##### Column Temperature Alteration

(**A**) A robustness test was carried out by increasing the column temperature (+5 °C). The effect on both retention time and peak area was evaluated for each component in the UGT2B17 assay as stated in Table 4. Optimization of the method was conducted on the HPLC system and 25 °C was found to be the optimum column temperature. Column temperatures of 20 and 30 °C were chosen for the robustness tests, as they lie within ±5 °C of the optimized column temperature, according to analytical ICH guidelines.

An ANOVA test with a single factor analysis was carried out to study the effect of column temperature on the peak area of each component. Statistically, no significant difference was evidenced between the peak area of each component at two different column temperatures (*p*-value = 0.006) and a variance of 6.944 with the significance level set at 0.05. Overall, changing the column temperature from 25 to 30 °C resulted in keeping the retention time and peak area of UGT2B17 components. The most convenient column temperature for this analytical method was 25 °C, since the resolution between salicylic acid and UGT2B17 enzyme peaks at 30 °C was (0.94 min), smaller than the resolution between salicylic acid and UGT2B17 enzyme peaks at 25 °C (1 min).

(**B**) An ANOVA test with a single factor analysis was carried out for the effect of a reduction in column temperature from 25 to 20 °C on the peak area of each component. Statistically, no significant difference was evidenced between the peak area of each component at these two different column temperatures (*p*-value = 0.006) with a variance of 6.944 and the significance level set at 0.05. Thus, the UGT2B17 assay was considered to be a robust method upon column temperature variation as shown in Table 5.

#### 2.3.3. Linearity and Range

##### Calibration Curve of Testosterone Glucuronide

Different solution concentrations of testosterone glucuronide (0, 10, 30, 40, 60, 80, and 100 μM) in 47% phosphate buffer at pH = 3.8 + 13% acetonitrile +40% methanol were added to 50 µM of phenacetin and injected into the HPLC instrument for the standard calibration curve construction. A testosterone glucuronide calibration curve (Figure S1) was constructed by mean peak area ratio versus the concentration of testosterone glucuronide using the Excel software 2010 system. The results showed good linearity for testosterone glucuronide (y = 0.0212x − 0.0204) (standard error = 0.017/intercept = −0.019), with R^2^ value of 0.999. The obtained linear regression coefficient fits within the analytical ICH guidelines (R^2^ > 0.99). The linear range for the testosterone glucuronide standard is between 10 and 100 μM.

##### Calibration Curve of Testosterone

Different solution concentrations of testosterone (0, 20, 50, 80, 100, 160, 240, and 320 μM) in addition to 50 µM of phenacetin at each testosterone concentration were injected into a HPLC instrument for standard calibration curve construction. A testosterone calibration curve (Figure S2) was constructed by mean peak area ratio versus the concentration of testosterone using the Excel software 2010 system. The results showed good linearity for testosterone (y = 0.0229x − 0.1076) (standard error = 0.044/intercept = −0.108), with R^2^ value of 0.999. The obtained linear regression coefficient fits within the analytical ICH guidelines (R^2^ > 0.99). The linear range for testosterone standard is between 20 and 320 μM.

#### 2.3.4. Limit of Detection and Limit of Quantitation (LOD and LOQ)

The results shown in Table 6 illustrate that both UGT2B17 substrate (testosterone) and its metabolite (testosterone glucuronide) are characterized by low detection and quantification limit values. Thus, these values fit within the analytical ICH guidelines.

#### 2.3.5. Precision

##### Testosterone Intra-Assay Precision

Three concentration levels (25, 100, 200 µM) of testosterone were injected into the HPLC instrument thrice (*n* = 3) for intra-assay precision determination purposes. The concentration of testosterone was calculated from testosterone calibration curve equation: y = 0.0284x − 0.2297 (standard error = 0.106/intercept = −0.226), (R^2^ = 0.999). Table 7 shows that the percentage error was less than 5% for each testosterone concentration level.

##### Testosterone Glucuronide Intra-Assay Precision

Three concentration levels (80, 40, 10 µM) of testosterone glucuronide were injected into HPLC instrument thrice (*n* = 3) for intra-assay precision determination purposes. The concentration of testosterone glucuronide was calculated from the testosterone glucuronide calibration curve equation: y = 0.0198x − 0.0556 (standard error = 0.027/intercept = −0.088), (R^2^ = 0.996). Table 8 shows that the percentage error was <5% at each testosterone glucuronide concentration level.

##### Testosterone Inter-Assay Precision

Three concentration levels (25, 100, 200 µM) of testosterone were injected into the HPLC instrument thrice (*n* = 3) for three consecutive days for inter-assay precision determination purposes. The concentration of testosterone was calculated from the testosterone calibration curve equation of days 1, 2 and 3: y = 0.0284x − 0.2297 (standard error = 0.106/intercept = −0.226), (R^2^ = 0.998) for day 1, y = 0.023x − 0.102 (standard error = 0.041/intercept = −0.092), (R^2^ = 0.999) for day 2, y = 0.0183x − 0.0275 (standard error = 0.045/intercept = −0.073), (R^2^ = 0.998) for day 3. An ANOVA test of two-factors with replication analysis was performed for testosterone calibration curves for inter day 1, 2, and 3 variations to calculate if the slopes and the intercepts for each calibration curve at each day were comparable. The F-finding (variation between sample mean/variation within the sample) = 1.54 (*p* < 0.01) with the significance level set at 0.05 and variance = 0.064. The F-finding is not much greater than 1; thus, this means that the variance between the samples is no greater than the variance within the sample and the sample probably comes from populations within the same mean. Table 9 shows that the percentage error was ≤10% at each testosterone concentration level.

##### Testosterone Glucuronide Inter-Assay Precision

Three concentration levels (10, 40, 80 µM) of testosterone glucuronide were injected into the HPLC instrument thrice (*n* = 3) for three consecutive days for inter-assay precision determination purposes. The concentration of testosterone was calculated from the testosterone calibration curve equation of days 1, 2 and 3: y = 0.0198x − 0.0556 (standard error = 0.027/intercept = −0.088), (R^2^ = 0.996) for day 1, y = 0.025x − 0.0572 (standard error = 0.028/intercept = −0.092), (R^2^ = 0.998) for day 2, y = 0.0162x − 0.0553 (standard error = 0.022/intercept = −0.090), (R^2^ = 0.996) for day 3. An ANOVA test of two-factors with replication analysis was performed for testosterone glucuronide calibration curves for inter day 1, 2, and 3 variations to show if the slopes and the intercepts for each calibration curve at each day are comparable. The F-finding (variation between sample mean/variation within the sample) = 0.325 (*p* < 0.01) with the significance level set at 0.05 and variance = 0.032, is smaller than 1. This shows that the theoretical concentrations at each day are close together (low variability) relative to the variability within each sample. Table 10 shows that the percentage error was <10% at each testosterone concentration level.

#### 2.3.6. Stability Test

##### Stability Test of Testosterone (Substrate of UGT2B17 Enzyme)

Stability tests of testosterone were examined in our previous study (Salhab, H. et al., 2019) for three different concentrations (25, 100, and 200 µM). Testosterone was relatively stable for four days at ambient temperature in natural light conditions. The percentage of recovery and accuracy fit within the acceptable analytical range (80–120%) for ICH guidelines for the three concentration of testosterone levels [10].

##### Stability Test of Testosterone Glucuronide (Metabolite of UGT2B17 Enzyme)

The stability of testosterone glucuronide was carried out for three low, medium, and high concentrations (10, 40, and 80 µM) for 72 h at ambient room temperature. An internal standard (Phenacetin) of 50 µM was added to each testosterone glucuronide concentration level batch. Each batch was injected into the HPLC instrument and analyzed thrice (*n* = 3). To determine the concentration of testosterone glucuronide at days 1, 2 and 3, a calibration curve of testosterone glucuronide was run at 0, 10, 15, 20, 40, 60 and 100 µM for days 1, 2 and 3. Table 11 and Figure S3 illustrate the outcomes of the testosterone glucuronide stability test.

The results illustrate that the concentration of testosterone glucuronide and the chromatographic peak area were consistent between Days 1, 2, and 3. Calibration curves were plotted for 0, 48, and 72 h, and thus, the average calibration curve equation for the three days was: y = 0.012x − 0.0134 (standard error = 0.004/intercept = −0.010), (R^2^ = 0.999) (Figure S4). Based on Figure S3 results, percentage accuracy and recovery fall within the acceptable ICH guidelines (80–120%). The results revealed that testosterone glucuronide was stable for three days at normal ambient conditions [12].

### 2.4. In Vitro Potential Assessment of UGT2B17 Enzyme Activity Inhibition by Salicylic Acid

**Figure 3:**
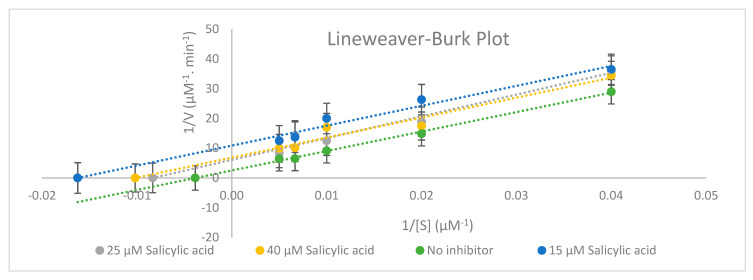
Representative Lineweaver–Burk plot for the inhibition of UGT2B17-catalysed testosterone glucuronide (testosterone (25–200 µM)) with 0, 15, 25, and 40 µM salicylic acid (*n* = 3).

To assess the potential UGT2B17 enzyme activity inhibition by salicylic acid in human supersomes, different salicylic acid concentrations levels (0, 15, 25, and 40 µM) were incubated at 37 °C with different concentrations of UGT2B17 substrate testosterone (25, 50, 100, 150, and 200 µM). The standard incubation mixture for each reaction tube contained: 2 mM of HPLC Uridine 5′-diphosphogluouronic acid trisodium salt (UDPGA), 25 μg/mL of alamethicin, 8 mM of magnesium chloride (MgCl_2_), and 0.067 M of phosphate buffer of pH = 7.4. The reaction was stopped at different specified timepoints, and the UGT2B17 assay incubation time was 60 min. Figure 3 and Table 12 show the in vitro potential assessment of UGT2B17 enzyme activity inhibition by salicylic acid in human supersomes.

The HPLC method was successfully optimized and validated for the purpose of quantifying testosterone glucuronide metabolite concentration in human supersomes. A UGT2B17 incubation assay in human supersomes has been optimized and correlated with protein concentration and incubation times, according to the previous study [2] (Figure S5 and Table S5). This report extends our previous studies, which revealed that CYP2C11 enzyme activity was inhibited non-competitively by salicylic acid [10], and CYP2E1 enzyme activity was inhibited competitively and non-competitively by salicylic acid [11].

Testosterone can be glucuronidated to testosterone glucuronide by means of the UGT2B17 enzyme [3]. This enzyme plays an important role in the glucuronidation process; however, the UGT2B15 enzyme was found to play a minor role in this process [2]. The rate of UGT2B17 enzyme glucuronidation was found to be doubled compared to the rate of UGT2A1 enzyme glucuronidation. Studies have shown that about 35% of marketed drugs are metabolized by Phase 2 enzymes via UGT-catalysed glucuronidation reactions [13]. Thus, this experimental work has made the first potential assessment of UGT2B17 enzyme activity inhibition by salicylic acid. Performing a UGT2B17 assay inhibition study with salicylic acid offers a clear key route to determining a risk assessment for drug–drug interactions. Therefore, UGT assays may be effective in assessing the risk of causing adverse drug reactions (ADRs) with other marketed drugs [14].

Interestingly, recent studies have reported that anti-cancer drugs such as Lapatinib and Imatinib can inhibit competitively UGT2B17 enzyme activity, thus causing drug–drug interactions, when UGT2B17 substrates are co-administrated [15]. Anabolic steroids such as testosterone and epitestosterone are cleared by the UGT2B17 isozyme [2]. Previous reports revealed that epitestosterone, ibuprofen and diclofenac act as a competitive inhibitor against UGT2B17 enzyme activity [2].

Previous work conducted by the authors revealed that CYP2C11 enzyme activity was inhibited non-competitively by salicylic acid [10], and CYP2E1 enzyme activity was inhibited competitively and non-competitively by salicylic acid [11]. Drugs that are substrates for the CYP2C11 enzyme have a low potential to cause drug interaction with salicylic acid in rats. Moreover, drugs that are substrates for the CYP2E1 enzyme could have both low and high abilities to cause drug interaction with salicylic acid in rats.

An ANOVA test of two factors with replication analysis was performed for the inhibition study. Statistically significant differences were evidenced between times and concentrations (*p* < 0.001) with significant interaction between the two (*p* = 0.001) with the significance level set at 0.05. This research (Figure 3 and Table 12) has demonstrated that UGT2B17 enzyme activity was inhibited uncompetitively by salicylic acid based on the in vitro inhibition study in human supersomes and the Lineweaver–Burk plot shape.

According to Table 12, 25 µM of salicylic acid can be considered as a saturation concentration since the K_m_ value (K_m_ = 119.90 µM) at 25 µM salicylic acid is greater than other K_m_ (K_m_ = 61.58 µM for 15 µM salicylic acid) and (K_m_ = 98.19 µM for 40 µM salicylic acid). Based on Table 12, the maximum velocity of the reaction (V_max_) reduced, and the Michaelis constant (K_m_) decreased from 0 to 40 µM salicylic acid. In fact, some experimental evidence has shown that drug–drug interactions occur more frequently with Phase 1 enzymes than Phase 2 enzymes [5].

This study will be beneficial for the in vivo study of salicylic acid in the healthcare screening domain and clinical trials. Moreover, it will give possible indications for its safe administration with other drugs. Since the UGT2B17 enzyme is responsible for the metabolism of salicylic acid in human supersomes, it is now important to assess the full effect of the UGT2B17 enzyme inhibition by salicylic acid in vivo.

## 3. Materials and Methods

### 3.1. Chemicals and Reagents

All HPLC analytical solvents (methanol and acetonitrile) were obtained from Sigma Aldrich, Co. (Gillingham, UK). High purity (>98%) salicylic acid, magnesium chloride (MgCl_2_), testosterone, phenacetin, monobasic and dibasic potassium phosphate, 85% purity phosphoric acid and Tris-HCL were obtained from Sigma Aldrich, Co. (Gillingham, UK). Finally, HPLC Uridine 5′-diphosphogluouronic acid, HPLC alamethicin from Trichoderma viride, and testosterone glucuronide were procured from Carbosynth limited (Compton, UK).

### 3.2. Human UGT2B17 Supersomes

The UGT2B17 enzymes in this study were purchased as UGT2B17 human supersomes from Corning International (One Riverfront Plaza, Corning, NY, USA) and kept at −80 °C. Human UGT2B17 supersomes were examined by the manufacturer for UGT content activity and protein concentration determination.

### 3.3. High Performance Liquid Chromatography (HPLC) Conditions

The mobile phase phosphate buffer at pH = 3.8 was prepared by using a 570 pH meter obtained from JENWAY limited (Staffordshire, UK). A MICROSTAR 17R high speed-centrifuging instrument and 1.5-mL centrifuging tubes were purchased from the VWR Company (Magna Park, Leicestershire, UK). UGT2B17 components were analyzed using a Shimadzu LC-2010A HT (200 UV-detector) system (Tokyo, Japan) equipped with a low-pressure quaternary pump and a degasser. Chromatographic separation of UGT2B17 components was achieved on a SUPELCO C18 column (25 cm × 4.6 mm, 5 µm particle size) and purchased from Merck (Old Brickyard, Gillingham, UK). Peak area for each component was analyzed by the HPLC Lab Solution 1 software system using the manual integration peak icon.

UGT2B17 components (UGT2B17 enzyme, salicylic acid, phenacetin, testosterone, and testosterone glucuronide) were separated using a low-pressure gradient elution system. The mobile phase was made up of: phosphate buffer (0.01 M) at pH = 3.8 (A), acetonitrile (B), and methanol (C). The mobile phase was as follows: (47% A: 13% B: 40% C) 0 min, (47% A: 13% B: 40% C) 9 min, (18% A: 13% B: 69% C) 9.01 min, (5% A: 13% B: 82% C) 17 min, (47% A: 13% B: 40% C) 17.01 min, (47% A: 13% B: 40% C) 20 min. The HPLC instrument was operated at 1 mL/min of flow rate, 243 nm UV detection, and the column was conditioned at 25 °C. The injection volume was set at 10 μL. The outcomes represent the σ (standard deviation) of triplicate values.

### 3.4. Potential Assessment of UGT2B17 Assay Inhibition by Salicylic Acid In Vitro

To determine the potential UGT2B17 activity inhibition by salicylic acid, the UGT2B17 metabolite (testosterone glucuronide) was quantified on the HPLC. UDP-regenerating system (2 mM uridine diphosphate glucuronic acid (UDPGA), 25 μg/mL alamethicin, and 8 mM magnesium chloride) (Figure S6 and Table S6) was used in evaluating testosterone glucuronide metabolism in the UGT2B17 assay. Each centrifuging tube was incubated using a UDP-regenerating system, 0.2 mg/mL of UGT2B17 supersomes, and 50 mM of Tris-HCL buffer (pH = 7.5). A range of testosterone concentrations (25, 50, 100, 150, and 200 μM) were added with a range of salicylic acid concentrations (0, 15, 25, and 40 µM) to each incubation tube, yielding a total of 500 μL volume in each tube. Each centrifuge tube was pre-incubated for 5 min in a water bath at 37 °C before the UDP-regenerating system addition. The percentage of organic solvent in each centrifuge tube did not exceed 1% *v*/*v*. The tubes were incubated for 60 min in a water bath at 37 °C. Phenacetin (50 μM, 300 μL) dissolved in ice-cold HPLC-grade acetonitrile was added to each incubation tube for reaction termination. Protein separation was obtained in each incubation tube after centrifuging at 13,000× *g* for 5 min using a micro centrifuge [16]. Finally, 500 μL of supernatant was collected from each tube and mixed with 300 μL of mobile phase in HPLC vials. The solution (10 μL) was injected for HPLC analysis.

### 3.5. Preparation of Analyte and Metabolite Standards

#### 3.5.1. Salicylic Acid and Testosterone Stock and Standard Solutions Preparation

Salicylic acid (1.38 mg) was weighed and dissolved in 50 mL of mobile phase yielding a stock solution concentration of 200 μM. Standard solutions of salicylic acid (15, 25, and 40 μM) were prepared from the stock solution by serial dilution. Testosterone (5.76 mg) was weighed and dissolved in 50 mL of mobile phase yielding a stock solution concentration of 400 μM. Standard solutions of testosterone (25, 50, 150, 200, and 300 μM) were prepared from the stock solution by serial dilution. Phenacetin powder (0.9 mg) was weighed and dissolved in 100 mL of HPLC-grade acetonitrile yielding 50 μM concentration.

#### 3.5.2. Testosterone Glucuronide Stock and Standard Solution Preparation

Testosterone glucuronide of 100 μM was prepared as a stock solution. Standard solutions of testosterone glucuronide (10, 15, 20, 35, 40, 60 and 80 μM) were prepared from the stock solution by serial dilution in 47% phosphate buffer at pH = 3.8 + 13% acetonitrile + 40% methanol.

### 3.6. Mobile Phase Composition

The starting mobile phase composition on the HPLC instrument was made up of 47% of phosphate buffer (0.01 M) at pH = 3.8, 13% of HPLC grade acetonitrile, and 40% of HPLC grade methanol.

### 3.7. Statistical Analysis

Calibration curves for both testosterone and testosterone glucuronide were constructed using Microsoft Excel 2010 software. The testosterone calibration curve represents the average peak area of testosterone versus the actual concentration of testosterone. The testosterone glucuronide calibration curve represents the average peak area of testosterone glucuronide versus the actual concentration of testosterone glucuronide. Microsoft Excel 2010 software was used to determine the average peak area ratio of testosterone and testosterone glucuronide by phenacetin peak area. All results are calculated as average ± error.

Statistical analyses for validation parameters (% error, % recovery, % accuracy, range, LOD, and LOQ) were examined using Microsoft Excel 2010 software. The concentration of testosterone glucuronide metabolite produced at different time intervals and different salicylic acid concentrations was determined from the calibration curve of testosterone glucuronide for UGT2B17 inhibition analyses. An ANOVA test of single factor and two-factors with replication was performed using a directional one-sample *t*-test with the significance level set at 0.05 to test for a significant difference between calibration curves on different days. Based on the standard error, SC, AIC values and Lineweaver–Burk plot shape, the type of UGT2B17 inhibition was uncompetitive inhibition. The best fit model was chosen as a non-linear regression model since the AIC value was low. The ΔAIC gives substantial evidence for the model (ΔAIC < 2).

The pharmacokinetic parameters (V_m_, K_m_, Cl_int_ (hepatic intrinsic clearance) were evaluated from a Lineweaver–Burk plot using the following equation:

$$V\;=\;\frac{\mathrm{Vmax}\;\times \;\left[S\right]}{\left[S\right]\;+\;\mathrm{Km}\;\left(1\;+\;\frac{\left[I\right]}{\mathrm{Ki}}\right)}$$

where V is the observed velocity, Vmax is the maximum rate of the reaction, S is the slope factor, Km is Michaelis constant, Ki is the inhibition constant, and I is the concentration of inhibitor.

## 4. Conclusions

In summary, the HPLC method for UGT2B17 assay was systematically developed using a mobile phase of phosphate buffer solution at pH = 3.8, methanol and acetonitrile in a gradient elution mode system. All analytical ICH parameters for UGT2B17 substrate (testosterone) and its metabolite (testosterone glucuronide) were systemically validated. The current study underlines the assessment of UGT2B17 enzyme activity inhibition by salicylic acid in human supersomes. Considering the above-listed data, testosterone glucuronide was found to be stable for 72 h in normal laboratory conditions. Nevertheless, our finding demonstrated that salicylic acid potentially inhibited UGT2B17 enzyme activity as a non-competitive inhibition mode in human supersomes.

These results provide guidance for a future in vivo study of salicylic acid in the healthcare screening domain or in clinical trials. More investigational in vivo studies should be done to evaluate the safety administration measures of taking salicylic acid with other marketed drugs. A common example where salicylic acid is taken with other drugs is in the case of colon cancer, where salicylic acid is taken with another FDA-approved drug such as Avastin or Bevacizumab, etc.

## Figures and Tables

**Table 1:** HPLC gradient elution programming for UGT2B17 enzyme assay.

Time (min)	Phosphate Buffer (pH = 3.8) (%)	Acetonitrile (%)	Methanol (%)
0.01	47	13	40
9.00	47	13	40
9.01	18	13	69
17.00	5	13	82
17.01	47	13	40
20.00	47	13	40

**Table 2:** Effect of flow rate on retention time and peak area of UGT2B17 components.

Flow Rate	UGT2B17 Components	Mean Retention Time (min)	Mean Peak Area (Mean ± σ)	Resolution
Gradient elution mode: normal conditions (flow rate = 1 mL/min)	UGT2B17 enzyme	2.19	58,891.67 ± 4.40	Better resolution obtained between five components (all compounds eluted earlier before 15 min).
	Salicylic acid (100 µM)	3.18	30,131.33 ± 1.72	
	Phenacetin (50 µM)	6.19	86,736.67 ± 2.50	
	Testosterone glucuronide (100 µM)	9.43	186,348.70 ± 1.03	
	Testosterone (200 µM)	14.40	442,266.30 ± 2.38	
Gradient elution mode at a flow rate of 0.8 mL/min	UGT2B17 enzyme	2.73	77,169.33 ± 0.36	Good resolution obtained between five compounds (testosterone eluted after 15 min).
	Salicylic acid (100 µM)	3.99	41,810.33 ± 5.30	
	Phenacetin (50 µM)	7.73	107,386.70 ± 1.64	
	Testosterone glucuronide (100 µM)	11.66	2,231,853.00 ± 5.02	
	Testosterone (200 µM)	15.73	537,723.00 ± 0.64	

**Table 3:** Effect of flow rate on retention time and peak area of UGT2B17 components.

Flow Rate	UGT2B17 Components	Mean Retention Time (min)	Mean Peak Area (Mean ± σ)	Resolution
Gradient elution mode: normal conditions (flow rate = 1 mL/min)	UGT2B17 enzyme	2.19	58,891.67 ± 4.40	UGT2B17 enzyme and salicylic acid were well separated (very good resolution) (difference in retention time = 1 min).
	Salicylic acid (100 µM)	3.18	30,131.33 ± 1.72	
	Phenacetin (50 µM)	6.19	86,736.67 ± 2.50	
	Testosterone glucuronide (100 µM)	9.43	186,348.70 ± 1.03	
	Testosterone (200 µM)	14.38	442,266.30 ± 2.38	
Gradient elution mode (flow rate = 1.2 mL/min)	UGT2B17 enzyme	2.73	45,843.33 ± 0.45	Good separation between UGT2B17 enzyme and salicylic acid (difference in retention time = 0.81 min).
	Salicylic acid (100 µM)	3.99	29,678.67 ± 1.62	
	Phenacetin (50 µM)	7.73	72,981.67 ± 2.19	
	Testosterone glucuronide (100 µM)	11.66	153,252.70 ± 1.24	
	Testosterone (200 µM)	15.73	383,111.00 ± 2.85	

**Table 4:** Effect of column temperature on retention time and peak area of UGT2B17 components.

Column Temperature	UGT2B17 Components	Mean Retention Time (min)	Mean Peak Area (Mean ± σ)	Resolution
Gradient elution mode at normal conditions (T = 25 °C)	UGT2B17 enzyme	2.19	58,891.67 ± 4.40	Very good separation for all components (resolution between UGT2B17 enzyme and salicylic acid was 1 min).
	Salicylic acid (100 µM)	3.18	30,131.33 ± 1.72	
	Phenacetin (50 µM)	6.19	86,736.67 ± 2.50	
	Testosterone glucuronide (100 µM)	9.43	186,348.70 ± 1.03	
	Testosterone (200 µM)	14.38	442,266.30 ± 2.38	
Gradient elution mode (T = 30 °C)	UGT2B17 enzyme	2.18	63,574.67 ± 4.19	Good separation between all components (resolution between UGT2B17 enzyme and salicylic acid was 0.94 min).
	Salicylic acid (100 µM)	3.12	30,386.33 ± 1.52	
	Phenacetin (50 µM)	5.92	92,263.33 ± 4.94	
	Testosterone glucuronide (100 µM)	8.54	178,039.00 ± 5.85	
	Testosterone (200 µM)	14.21	456,677.30 ± 2.47	

**Table 5:** Effect of column temperature on retention time and peak area of UGT2B17 assay.

Column Temperature	UGT2B17 Components	Mean Retention Time (min)	Mean Peak Area (Mean ± σ)	Resolution
Gradient elution mode at normal conditions (T = 25 °C)	UGT2B17 enzyme	2.19	58,891.67 ± 4.40	All compounds were well separated (very good resolution)
	Salicylic acid (100 µM)	3.18	30,131.33 ± 1.72	
	Phenacetin (50 µM)	6.19	86,736.67 ± 2.50	
	Testosterone glucuronide (100 µM)	9.43	186,348.70 ± 1.03	
	Testosterone (200 µM)	14.38	442,266.3 ± 2.38	
Gradient elution mode (T = 20 °C)	UGT2B17 enzyme	2.20	61,039.00 ± 0.19	Good separation between all components
	Salicylic acid (100 µM)	3.25	28,490.33 ± 0.95	
	Phenacetin (50 µM)	6.46	85,718.67 ± 3.15	
	Testosterone glucuronide (100 µM)	10.39	175,511.00 ± 4.88	
	Testosterone (200 µM)	14.56	426,661.30 ± 0.23	

**Table 6:** LOD and LOQ for testosterone and testosterone glucuronide.

Analytes	Testosterone	Testosterone Glucuronide
Limit of Detection (LOD)	6.42 μM	2.76 μM
Limit of Quantitation (LOQ)	19.46 μM	8.38 μM

**Table 7:** Testosterone intra-assay precision data.

Testosterone Standard	Theoretical Concentration (µM)	Standard Deviation (std)	Percentage Error (% error)
Low activity (C = 25 µM)	26.44	0.43	1.64
Medium activity (C = 100 µM)	95.53	1.19	1.24
High activity (C = 200 µM)	178.67	4.09	2.29

**Table 8:** Testosterone glucuronide intra-assay precision data.

Testosterone Glucuronide Analyte	Theoretical Concentration (µM)	Standard Deviation (std)	Percentage Error (% error)
Low activity (C = 10 µM)	11.45	0.27	2.35
Moderate activity (C = 40 µM)	39.19	0.57	1.45
High activity (C = 80 µM)	75.24	0.91	1.20

**Table 9:** Testosterone inter-assay precision data.

Testosterone Analyte		Average Peak Area (*n* = 3 Each Level)	Theoretical Concentration (µM)	Standard Deviation (std)	Percentage Error (% Error)
Low activity (C = 25 µM)	Day 1	0.51	23.78	1.65	6.93
	Day 2	0.44			
	Day 3	0.37			
Medium activity (C = 100 µM)	Day 1	2.42	96.32	2.34	2.42
	Day 2	2.13			
	Day 3	1.78			
High activity (C = 200 µM)	Day 1	4.97	201.00	20.09	10.00
	Day 2	4.32			
	Day 3	4.16			

**Table 10:** Testosterone glucuronide inter-assay precision data.

Testosterone Glucuronide Analyte		Average Peak Area (n = 3 Each Level)	Theoretical Concentration (µM)	Standard Deviation (std)	Percentage Error (% Error)
Low activity (C = 10 µM)	Day 1	1.43	11.08	0.31	2.82
	Day 2	1.74			
	Day 3	1.05			
Medium activity (C = 40 µM)	Day 1	0.72	37.35	1.30	3.49
	Day 2	0.86			
	Day 3	0.53			
High activity (C = 80 µM)	Day 1	0.17	71.88	2.79	3.88
	Day 2	0.21			
	Day 3	0.13			

**Table 11:** Stability test outcome of testosterone glucuronide.

Stability Test Parameters	Testosterone Glucuronide Actual Concentration (µM)			
		10	40	80
Theoretical concentration (µM)	0 h	9.44	37.78	72.17
	48 h	9.44	37.78	74.04
	72 h	9.29	37.67	73.08
% Recovery ^a^	48 h	100.00	100.00	102.59
	72 h	98.42	99.72	101.26
Accuracy ^b^ (%)	0 h	105.58	105.56	109.79
	48 h	105.58	105.56	107.45
	72 h	107.08	105.83	108.65

^a^ % Recovery = ((theoretical concentration of testosterone glucuronide at Day 2)/theoretical concentration of testosterone glucuronide at Day 1) × 100. ^b^ Accuracy = 100 − ((theoretical concentration − actual concentration)/actual concentration) × 100.

**Table 12:** Pharmacokinetic parameters of UGT2B17 inhibition study. Values are noted as mean ± σ (n = 3) (*p* = 0.001) at α = 0.05.

Pharmacokinetic Parameters	0 µM Salicylic Acid	15 µM Salicylic Acid	25 µM Salicylic Acid	40 µM Salicylic Acid
K_m_ (µM)	259.74 ± 2.14	61.58 ± 0.068	119.90 ± 0.99	98.19 ± 0.81
V_max_ (µM^−1^ min^−1^)	0.40 ± 1.77	0.092 ± 0.86	0.16 ± 0.73	0.15 ± 0.65
Cl_int_ (µM^−2^ min^−1^)	0.0015 ± 4.48	0.0015 ± 0.30	0.0014 ± 5.07	0.0015 ± 4.66

## Data Availability

The data presented in this study are available in this article.

## Supplementary Materials

The following are available online, Figure S1: Standard calibration curve of testosterone glucuronide. Mean peak area ratio is equal to mean peak area of the analyte (*n* = 3) divided by the mean peak area of the internal standard phenacetin (*n* = 3), Figure S2: Standard calibration curve of testosterone, Figure S3: Stability test results of testosterone glucuronide at 0, 48, and 72 h (*n* = 3). Dashed lines: represent the acceptable range of the percentage recovery according to ICH guidelines. Solid lines: represent the stability test of testosterone glucuronide at 10, 40, and 80 µM for 0, 48, and 72 h, Figure S4: Average standard calibration curve of testosterone glucuronide, Table S5: Linear regression, linear range, limit of detection, and limit of quantitation analysis for UGT2B17 assay incubation, Figure S5: Calibration curve of testosterone glucuronide, Table S6: 60 min incubation assay of UGT2B17 enzyme activity (*n* = 3) in human supersomes, Figure S6: Bar chart for the formation of testosterone glucuronide metabolite after 60 min of incubation mixture.

## Sample Availability

Samples of the compounds are not available from the authors.
