# Supplementary material for: Potential Assessment of UGT2B17 Inhibition by Salicylic Acid in Human Supersomes In Vitro
Source: Molecules. 2021 Jul 21;26(15):4410. doi: 10.3390/molecules26154410 (PMC8348562; doi:10.3390/molecules26154410)
Supplement: Supplementary file 1 [file molecules-26-04410-s001.zip › molecules-1271345-supplementary.pdf]

Supplementary Information

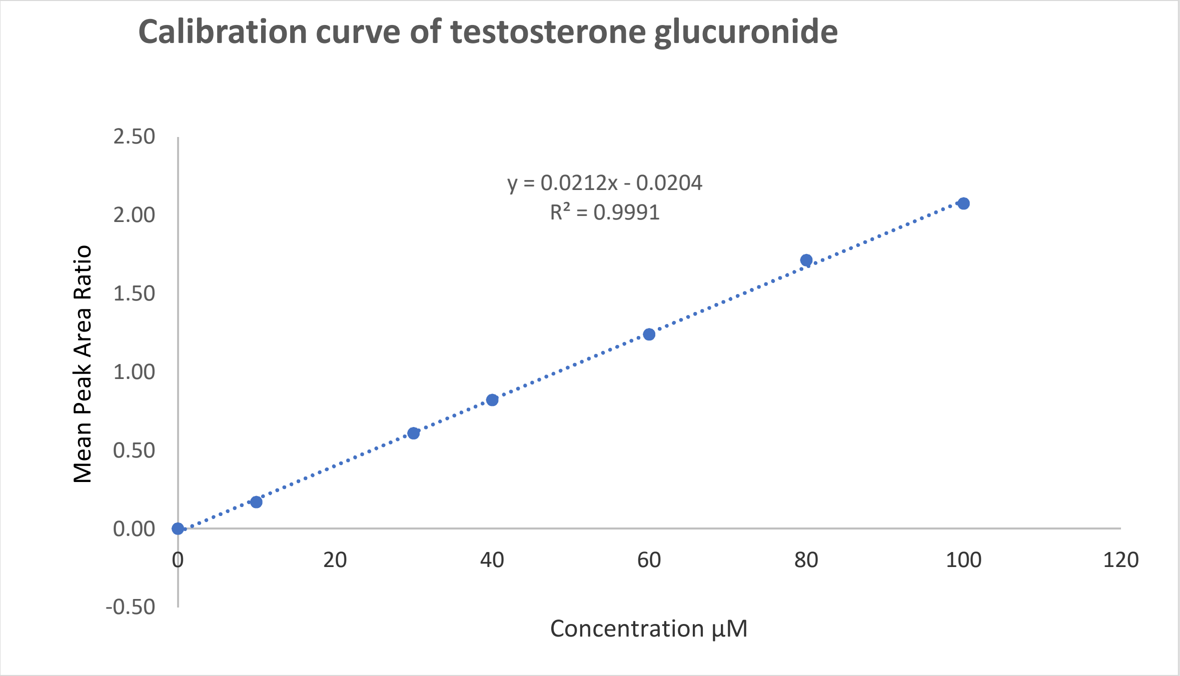

Figure S1. Standard calibration curve of testosterone glucuronide. Mean peak area ratio is equal to mean peak area of the analyte (n=3) divided by the mean peak area of the internal standard phenacetin (n=3).

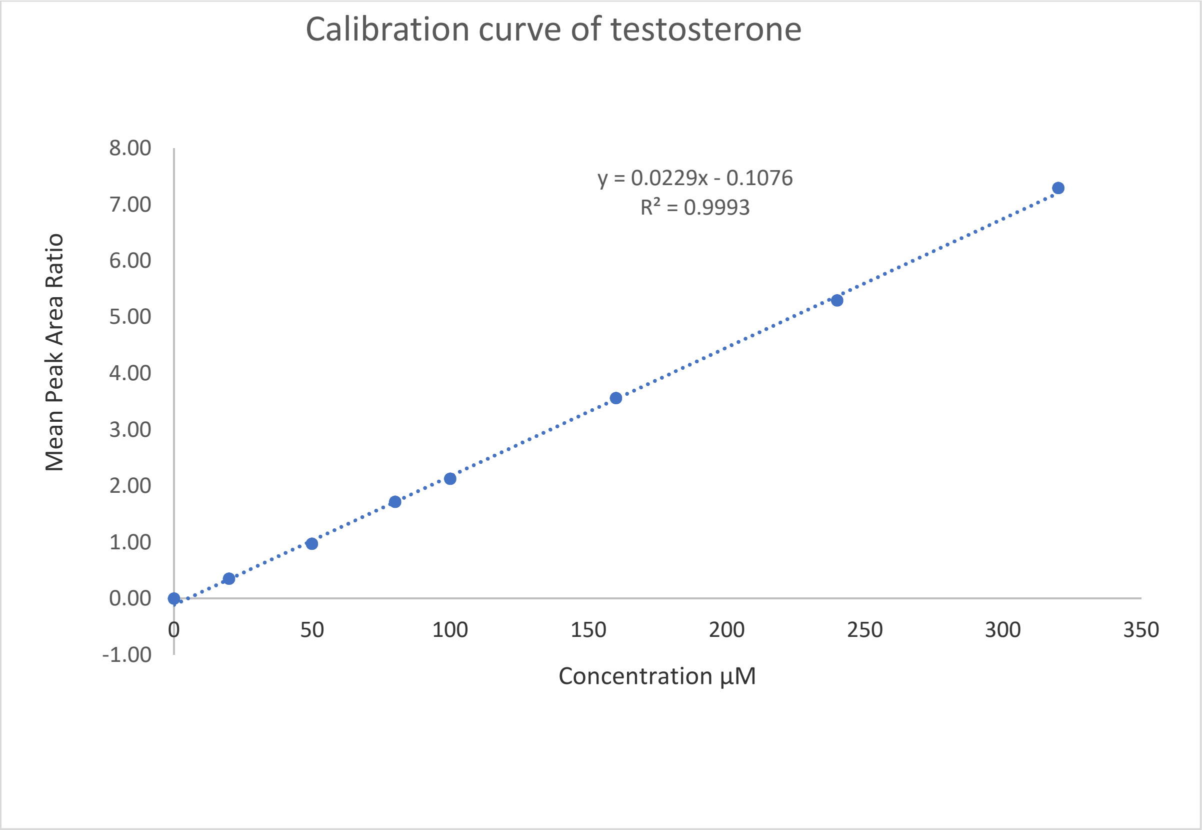

Figure S2. Standard calibration curve of testosterone.

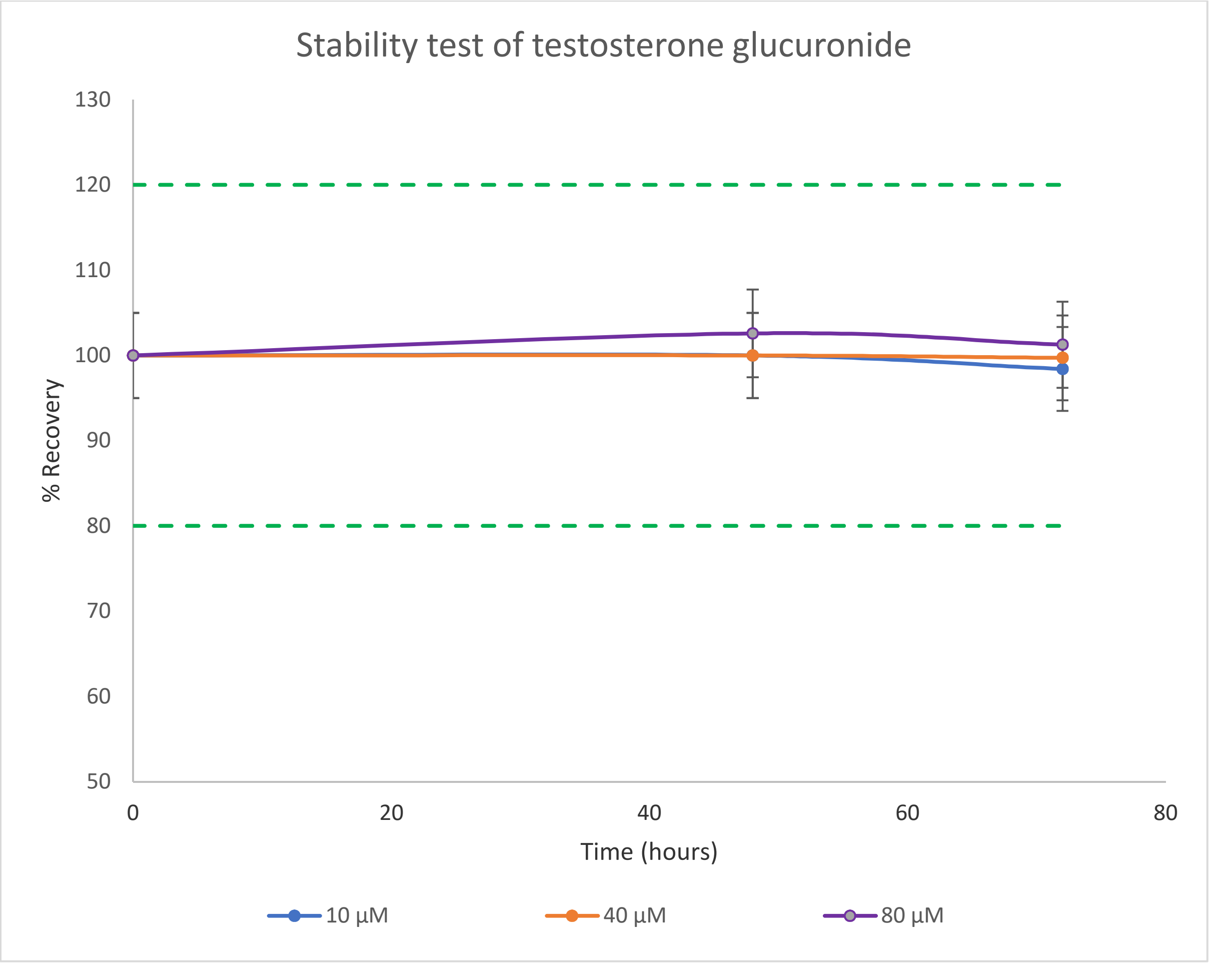

Figure S3. Stability test results of testosterone glucuronide at 0, 48, and 72 hours (n=3). Dashed lines: represent the acceptable range of the percentage recovery according to ICH guidelines. Solid lines: represent the stability test of testosterone glucuronide at 10, 40, and 80 µM for 0, 48, and 72 hours.

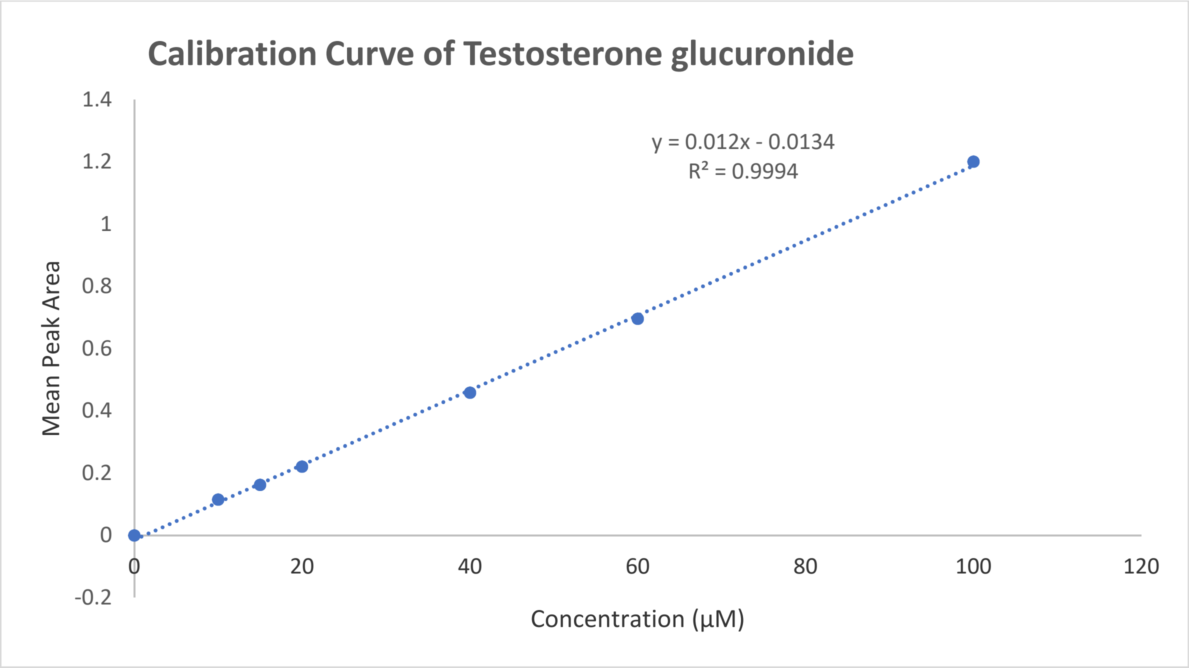

Figure S4. Average standard calibration curve of testosterone glucuronide.

UGT2B17 incubation of 60 mins:
Different concentrations of testosterone (400, 300, 200, 150, 100, 50 and 25 µM) were used in UGT2B17 incubation mixture at 60 mins of incubation time using 2 mM UDPGA, 25 µg/mL alamethicin, 50 mM Tris-HCl at pH=7.5, and 8 mM Magnesium chloride.

Table S5 shows the regression analysis on calibration curves and limit of detection (n=3) for testosterone glucuronide (metabolite of UGT2B17 enzyme) for the UGT2B17 assay incubation.

Components	Regression equation (y=ax+b)[a]	R^2	Linear range (µM)	LOD (µM)	LOQ (µM)
Testosterone glucuronide	y=0.0146x-0.0017	0.9996	10-100	1.4274	4.3253

Table S5 Linear regression, linear range, limit of detection, and limit of Quantitation analysis for UGT2B17 assay incubation.

x is the concentration of the compound in the reaction mixture (µM), y is the peak area ratio of the standard over the peak area of the internal standard (Phenacetin 50 µM), a is the slope and b is the y-intercept of the linear regression.
The analytical range covers the concentration range of the obtained quantitative results without the recalibration needs, which is performed to evaluate both upper and lower limits of the working range. The regression line for testosterone glucuronide metabolite (R^2 = 0.9996) in **Table S5 and Figure S5** demonstrates a linear range of 10 – 100 µM.

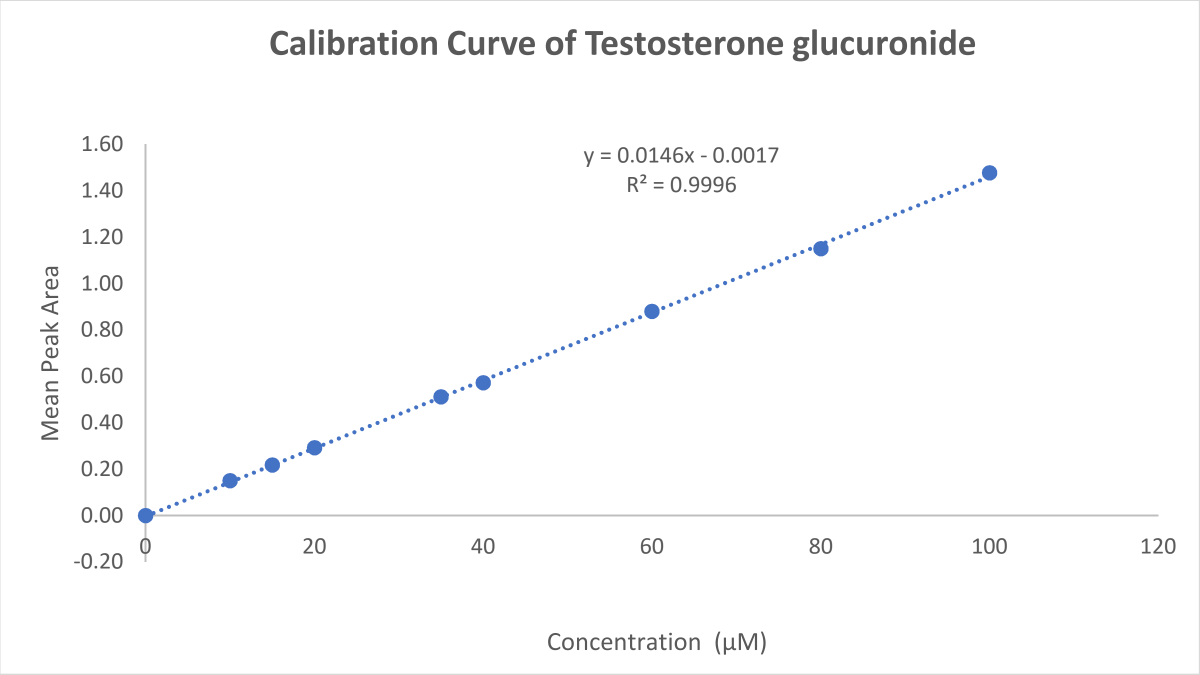

Figure S5 : Calibration curve of testosterone glucuronide

Table S6 illustrates the concentration of testosterone glucuronide formed after the incubation in UGT2B17 human supersomes incubation assay (n=3).

Probe substrate	Amount before incubation (μM)	Metabolite	Amount after incubation (μM)
Testosterone	400	Testosterone glucuronide	5.74
	300		8.19
	200		8.71
	150		11.00
	100		8.31
	50		6.20
	25		3.56

Table S6 Outcomes of UGT2B17 human supersomes incubation assays (n=3) of 60 mins incubation using different range of testosterone concentrations.

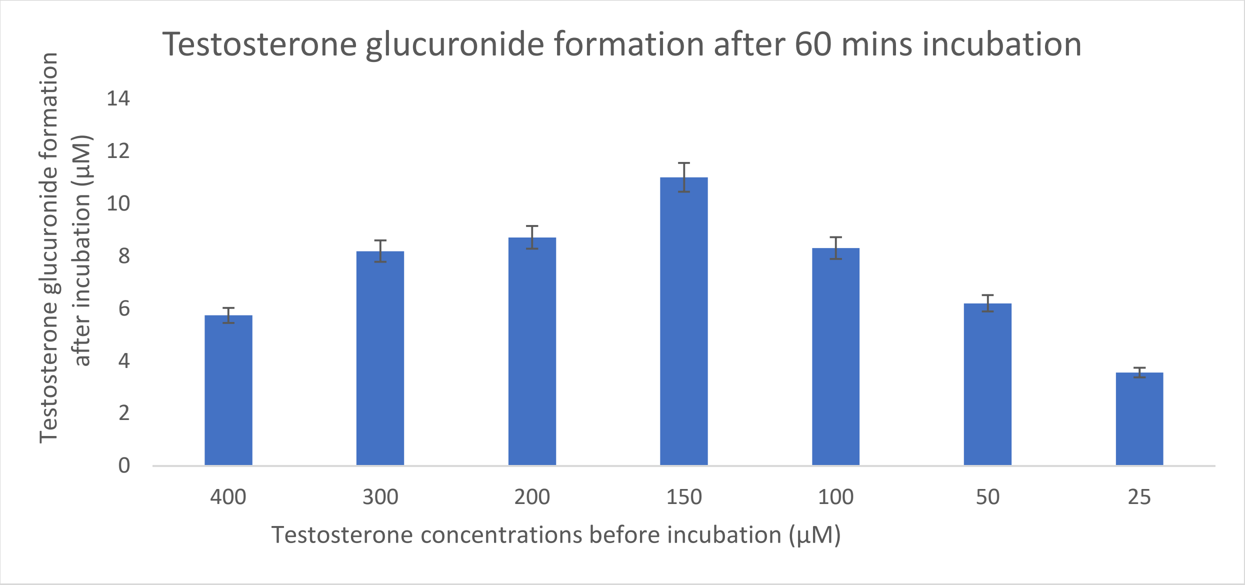

***Figure S6** Bar chart shows the formation of testosterone glucuronide after 60 mins of UGT2B17 assay incubation at different testosterone concentration levels (25, 50, 100, 150, 200, 300 and 400 µM).*

Table S6 and **Figure S6**, shows that testosterone at concentrations of 200 and 150 µM (before incubation) metabolized to testosterone glucuronide at its maximum amount (8.71 and 11.00 µM) compared to other different testosterone concentrations level (400, 300, 50 and 25 µM). Apparently, at 200, and 150 µM concentrations, a formation of testosterone glucuronide was produced at its maximal limit. Thus, in this study, salicylic acid of different concentrations will be incubated with testosterone at different concentrations stated (200, 150, 100, 50 and 25 µM).
